# Factors Influencing Survival in Carcinoma of the Ovary

**DOI:** 10.1038/bjc.1971.30

**Published:** 1971-06

**Authors:** Jennifer L. Dyson, J. O. W. Beilby, S. J. Steele

## Abstract

**Images:**


### FACTORS INFLUENCING SURVIVAL IN CARCINOMA OF THE OVARY

JENNIFER L. DYSON, J. O. W. BEILBY AND S. J. STEELE

*From the Bland-Sutton Institute of Pathology and Department of Obstetrics and Gynaecology, The Middlesex Hospital, London, W.1*

Received for publication March 18, 1971

**SUMMARY.—Three hundred and nineteen patients with primary adenocarcinoma of the ovary were studied to define those factors, many of them histopathological, which influence survival. The paper considers the stage of spread at the time of operation, the histological type of the tumour, its grade and in particular its mitotic activity, which proved a significant feature *per se* in assessing prognosis in ovarian cancer.**

A SURVEY has been carred out on 319 patients with primary adenocarcinoma of the ovary to evaluate some of the factors which influence survival in this disease. It became apparent during the investigation that the mitotic count was of considerable prognostic significance. Other factors considered were the histological type of the tumour, the stage to which it had spread at the time of operation and its histological grade.

Ford (1928) was the first person to evaluate the prognosis according to the stage of spread at the time of operation, and the first large series was published in 1932 (Heyman, 1932). The first paper on grading appeared at much the same time (Taylor, 1929), and most series since that of Pemberton (1940) have treated it as an important prognostic factor.

Although cellular atypicality, as a feature of malignancy, has been discussed frequently in relation to grading, mitotic activity has received much less attention. Barzilai (1943) reported that mitoses were " frequently seen " in the group of tumours which she called " sero-anaplastic ", and Allan and Hertig (1949) used profusion of mitoses as one of the indicators of the most malignant neoplasms. But at the time when this study was undertaken, no publication had been found in which mitoses were considered as a separate feature. The only published work specifically devoted to this point was by Novak and Woodruff (1967) after the present investigation had been largely completed. It confirms that the count of mitoses per high power field is of prognostic significance in carcinoma of the ovary.

### MATERIALS AND METHODS

The material for this survey came from 319 patients at four hospitals.

| Hospital | Years | Cases |
|---|---|---|
| The Middlesex Hospital, London | 1946–62 | 138 |
| The Hospital for Women, Soho Square, London | 1943–62 | 94 |
| The Hospital for Women, Chelsea, London | 1956–61 | 53 |
| The Whittington Hospital, London | 1952–61 | 34 |

Requests for reprints to J. O. W. Beilby at the Bland-Sutton Institute, Middlesex Hospital.

*Criteria for selection.*—Since it was decided to make this series as homogeneous as possible, patients were included only if they had a primary adenocarcinoma of the ovary. Tumours metasatic to the ovary or whose origin was doutbful were excluded, and biopsy material was acceptable only where the operation notes made it clear that the surgeon had seen a distinct ovarian mass which he believed to be the primary tumour. (The importance of strict criteria for the inclusion of biopsy material has been argued by Meigs, 1940.) Granulosa cell tumours, theca cell tumours, arrhenoblastomas, dysgerminomas and malignant teratomas have a different behaviour and age distribution and have not been considered here.

The patients studied had all had an operation or a biopsy which they survived by at least 1 month, and all were followed up for 5 years or until they died of their disease. Four hundred and ninety-three cases were originally examined and 174 were rejected because they did not fulfil the criteria set out, or because the notes or histological material were inadequate.

*The stage of spread at the time of operation.*—The following system of staging was used:

| | |
|---|---|
| Stage IA. | Unilateral growth which had not penetrated the ovarian capsule, including those malignant cysts ruptured at operation. |
| Stage IB. | Unilateral growth which had penetrated the ovarian capsule. |
| Stage IIA. | Bilateral growth, in which the ovarian capsule was intact on both sides. |
| Stage IIB. | Bilateral growth, in which the ovarian capsule had been penetrated, but spread elsewhere was confined to the genital tract. |
| Stage IIIA. | Spread to the pelvic peritoneum or viscera, but all macroscopically visible tumour resected. |
| Stage IIIB. | Inoperable tumour confined to the pelvic cavity. |
| Stage IV. | Inoperable tumour with spread above the pelvic brim. |

*The histological type of the tumour.*—The tumours were typed histologically into three main groups; serous, mucinous and unclassified. Among the serous tumours have been included " mesonephromas " of the adult type and endometrioid tumours. The criteria for the diagnosis of endometrioid carcinomas have been laid down by Long and Taylor (1964), but the reported incidence of these tumours in various series ranging from from 4·8 per cent (Malloy, Dockerty, Welch and Hunt, 1965) through 11 per cent (Schueller and Kirol, 1966), 16·7 per cent (Long and Taylor, 1964) to 24 per cent (Santesson, quoted by Long and Taylor, 1964): confirms our experience that it is impossible to discriminate between the poorly differentiated serous and endometrioid carcinomas. The well differentiated endometrioid carcinomas, especially when they show squamous metaplasia, are readily identifiable (Fig. 1), but the incidence and behaviour of endometrioid carcinomas, throughout the whole range of differentiation, has proved impossible to quantitate.

A recent review of " Mesonephromas " of the clear cell type or those with a characteristic tubular pattern (Scully and Barlow, 1967) has given considerable plausibility to the concept that these represent a variant of endometrioid carcinoma and their existence as a distinct entity has also been disputed by Stowe (1955)

and Willis (1967). In any event other series (Parker, Dockerty and Randall, 1960; Novak and Woodruff, 1959; Czernobilsky, Silverman and Enterline, 1970) suggest that their behaviour is that of serous carcinomas.

Unclassified tumours have been included in this series. There histological appearance suggests that they are the anaplastic variants of the common serous and endometrioid, but not mucinous, carcinomas; and this belief, which is held by other workers (Taylor, 1959; Montgomery, 1948), has even led some (*e.g.* Barzilai, 1943) to use the term " sero-anaplastic " to describe them.

*The histological grade of the tumour.*—In the histological grading of carcinoma of the ovary problems which are inherent in any system of grading arise in relation to sampling. But differences from one part of the tumour to another are not usually marked, as the detailed study of Taylor and Long (1955) demonstrated, and it is our experience that any one tumour tends to have a consistent growth pattern which is reproduced in different parts of the tumour, and to a lesser extent in metastases.

The tumours were placed in three grades, depending on their microscopic pattern:

Grade 1. Well differentiated carcinomas, including cystic tumours with small areas of malignant proliferation in an otherwise benign epithelial lining.

Grade 2. Active tumours with invasion, showing complex papillary proliferation, but no solid growth.

Grade 3. Tumours showing areas of solid growth, sometimes with necrosis.

This classification led to the inclusion of relatively more tumours in Grade 3 than in other series (Allan and Hertig, 1949; Kent and McKay, 1960). But the inclusion of only " the wildly anaplastic " (Allan and Hertig, 1949) tumours in Grade 3 or the establishment of a separate Grade 4 for the anaplastic tumours (Van Orden, McAllister, Zerne and Morris, 1966) is hardly justified. Their behaviour is no worse than the equally lethal and much commoner tumours included in Grade 3 in this series.

One feature which, on this system of grading, could lead to error, is the misinterpretation of solid islands of squamous epithelium in adenoacanthomas (Fig. 2). Following the lead of others who have discussed the presence of squamous metaplasia as an indication of high grade malignancy (Pemberton, 1940; Malloy *et al.*, 1965), solid masses of squamous epithelium were initially regarded in this survey as a sign of a high level of malignancy, but it became clear that this was erroneous, and that the malignant potential—as in the uterine adenoacanthoma (Novak and Nally, 1957) lies in the glandular epithelium.

*Mitotic activity.*—The number of mitoses per high power field was counted in the most active area of each tumour (at a magnification of 420 times). In most instances an average count over at least six fields was taken. Occasionally, fewer fields had to suffice, for example in a highly necrotic tumour in which there was only a rim of viable tissue, and also in tumours in which there was only a small area of malignant change in an otherwise benign cyst.

In some tumours with an extensive fibrotic stroma, a large part of every high power field was occupied by fibrous tissue. In these cases an estimate was made of the number of mitoses there would have been in a cellular field, in order to give the same percentage of mitoses. This is the method advocated and adopted by

Bloom and Richardson (1957) in dealing with the similar problem of grading carcinoma of the breast.

### RESULTS AND DISCUSSION

Of the 319 women in this survey, 205 died, within 5 years of operation, and 114, or 35·7 per cent, survived for 5 years. Carcinoma of the ovary is not usually a disease with a protracted course; over half the patients who succumbed did so within 14 months of operation.

A wide variation in 5-year survival figures for carcinoma of the ovary has been quoted by Munnell and Taylor (1949), ranging from 6·3 per cent (Walter, Bachman and Harris (1941) to 65 per cent Counseller (1940). Table I summarises some recent series and shows that a 5-year survival rate of between 20 and 40 per cent is the usual finding.

There are three main reasons for variations in survival figures: differences in treatment, differences in diagnosis and differences in the selection of cases.

The claims made for different forms of treatment have been excellently reviewed by Rubin, Grise and Terry (1962), who point out that the efficacy of radiotherapy or different forms of chemotherapy remains a matter for dispute.

The diagnosis of malignancy in ovarian tumours is often a matter of considerable difficulty, and this led Taylor (1929) to apply the term " semi-malignant " to certain serous neoplasms. In the case of mucinous tumours, as pointed out by Saxen and Hakama (1964), assessment may be even more contentious, despite the work of Cariker and Dockerty (1954) in defining the criteria of malignancy.

Differences in diagnosis are not the only reasons for variation in survival figures. Different series are frequently compared on the unstated assumption that the patients comprise similar populations, but a brief review shows how unfounded this assumption is. Table I shows wide variations between the number of inoperable cases included in each series.

TABLE I.—*Some Previous Series of Carcinoma of the Ovary*

| Authors | Year | Cases | Percentage 5-year survival | Operability |
|---|---|---|---|---|
| Keettel, Fox, Longnecker and Latourette | 1966 | 248 | 24·9 | 153 inoperable |
| Pomerance, Moltz and Hall | 1966 | 124 | 27 | 89 inoperable |
| Van Orden, McAllister, Zerne and Morris | 1966 | 137 | 23 | 102 inoperable |
| Guerriero and Spiedel | 1963 | 92 | 30 | 57 Stage III and IV* |
| Ratzkovski and Hochman | 1963 | 70 | 21 | |
| Stone, Weingold, Sall and Sonnenblick | 1963 | 131 | 20·6 | |
| Platt, Rubenstone and Hirsch | 1962 | 89 | 35 | 63 Stage III and IV* |
| Kent and McKay | 1960 | 349 | 36·4 | 170 Stage III and IV* |
| Cutler, Ederer, Griswold and Greenberg | 1960 | 2250 | 24 | |
| Turner, ReMine and Dockerty | 1959 | 164 | 54·3 | |
| Carlin and Frodey | 1957 | 137 | 23·3 | 91 inoperable |
| Henderson and Bean | 1957 | 336 | 18·1 | 195 inoperable |
| Munnell, Jacox and Taylor | 1957 | 148 | 27 | 81 Stage III and IV* |
| Davis, Latour and Philpott | 1956 | 202 | 37·6 | 109 Stage III and IV* |
| Gardiner and Slate | 1956 | 96 | 32·3 | 67 inoperable |
| Kerr and Elkins | 1954 | 190 | 31·5 | 91 operable |

* Tumours with spread staged anatomically. In some stage III cases all visible tumour will have been resected at operation, but the majority of stage III cases, and all stage IV cases are inoperable.

In the current survey, the selection of cases undoubtedly operated in favour of high survival figures, for all patients with carcinomatosis peritonei and an uncertain primary, and all patients who died within a month of operation, were excluded. Furthermore, hospital populations vary, because general hospitals, specialist hospitals, and hospitals with radiotherapy departments are likely to attract different types of patient. It was noticeable in this survey that the Middlesex and Whittington hospitals and the Hospital for Women Soho Square had comparable survival figures of 30 to 36 per cent, whereas the survival at 5 years at the Chelsea Hospital was 53 per cent. However, in the Chelsea Hospital data, only 28 per cent of the patients had inoperable carcinomas, compared with the average of 41 per cent at the other hospitals. One may surmise that similar selection factors operate at the Mayo Clinic, from which a 54 per cent survival has been reported (Turner *et al.*, 1959).

*The stage of spread reached at the time of operation*

Table II shows the percentage survival in relation to the stage reached at operation.

TABLE II.—*Percentage Survival in Relation to Stage Reached at Operation*

| Stage | Operation | 5 years | Percentage living at 5 years |
|---|---|---|---|
| IA | 106 | 69 | 62 |
| IB | 35 | 17 | 49 |
| IIA | 14 | 6 | 43 |
| IIB | 20 | 7 | 35 |
| IIIA | 21 | 6 | 29 |
| IIIB | 30 | 4 | 13 |
| IV | 93 | 5 | 5 |

It is apparent that there are two outstanding groups. The 106 patients in stage IA with unilateral intracapsular lesions, and the group of 93 patients where the tumour had spread beyond the pelvis and was inoperable. The greatest differences in the stages occur between IA and IB, on the one hand, and IIIA and IIIB on the other. These correspond to the differences between unilateral tumours confined within the ovary, IA, and those which have spread to the surface of the ovary, IB, whilst the difference between stages IIIA and IIIB, represents those cases in which all macroscopically visible tumour was resected and those in which residual tumour had to be left at the time of operation.

To correspond to these divisions, the tumours were regrouped in three stages:

| | |
|---|---|
| Unilateral intracapsular tumours—Stage IA: | 106 cases |
| Operable tumours with spread beyond the ovarian capsule— Stage IB to IIIA: | 90 cases |
| Inoperable carcinomata—Stages IIIB and IV: | 123 cases |

The differences in survival between these three groups at 5 years are highly significant ($\chi^2 = 84$, $n = 2$, $P < 0{\cdot}001$). They are expressed as percentages in Fig. 3.

The theoretical advantages in using an anatomical basis for staging have been very ably argued by Van Orden and her colleagues (1966). It leads to an objective approach which does not depend on variations in surgical policy, and makes

FIG. 3.—The percentage in each group surviving at yearly intervals, according to the stage the tumour at operation.

comparison between cases more exact. If carcinoma of the ovary were a highly radiosensitive tumour, anatomical considerations would clearly play a greater part in staging, but the results show that the patient's survival depends to such a large extent on the presence or otherwise of residual tumour, that a method of staging based on operability seemed more appropriate.

The presence of ascitic fluid at operation was not taken into account in this survey, although it is generally held to be important (*e.g.* Platt *et al.*, 1962; Van Orden *et al.*, 1966). Malloy and his colleagues (1965) used a measure of more than 250 ml. of ascitic fluid present at operation, but in a retrospective study dealing with cases where ascites was not always considered important at operation, such precision could not be achieved, and it was thought better to omit a matter on which no accurate assessment could be made.

A separate investigation was carried out to determine the appropriate stage of unilateral intracapsular tumours which had ruptured during removal. There were 18 patients in this group of whom 12 (two-thirds) survived 5 years. This is almost identical with the 65 per cent 5 year survival of the 88 women with unilateral intracapsular lesions which had not ruptured. Such a finding has been reported before (*e.g.* Malloy *et al.*, 1965; Munnell *et al.*, 1957), but remains surprising, in view of the additional risk of dissemination of malignant cells.

---

EXPLANATION OF PLATES

FIG. 1.—A well differentiated endometroid carcinoma showing areas of squamous metaplasia. H. & E. × 100.

FIG. 2.—Solid islands of squamous epithelium in an adenoacanthoma. H. & E. × 100.

FIG. 5.—A serous carcinoma of high grade and numerous mitoses. H. & E. × 420.

FIG. 6.—A serous carcinoma very similar in pattern to that in Fig. 5 but with one mitosis per high power field; both this and the previous figure illustrate unilateral tumours from women in the eighth decade. The former died of recurrent tumour, whereas the latter was alive 10 years later. H. & E. × 420.

1

2

Dyson, Beilby and Steele

5

6

*Histological type of tumour*

In this survey the incidence of the various types of tumour was:

| | | |
|---|---|---|
| Serous | 185 | 58 per cent |
| Mucinous | 68 | 21 per cent |
| Unclassified | 66 | 21 per cent. |

This is the pattern of distribution found in most series (Saxen and Hakama, 1964), and may be taken as representative.

Fifty-three out of the 68 patients with mucinous tumours survived 5 years, whereas only 49 of the 185 with serous tumours, and 12 of the 66 with unclassified tumours did so. The much greater likelihood of survival for those having mucinous tumours than for those with serous tumours is statistically highly significant ($\chi^2 = 54{\cdot}5, n = 1, P < 0{\cdot}001$). On the other hand, although a smaller proportion of patients with unclassified tumours survived 5 years than did those with serous tumours, the difference is not significant ($\chi^2 = 1{\cdot}82$, $n = 1$, $0{\cdot}2 > P > 0{\cdot}1$).

TABLE III.—*The Total Number of Cases in Each Stage with, in Brackets, the Numbers Surviving at 5 Years*

| Type | Unilateral intracapsular IA | Operable IB, IIA, IIB, IIIA | Inoperable IIIB, IV |
|---|---|---|---|
| Mucinous | 43 (37) | 14 (11) | 11 (5) |
| Serous | 41 (28) | 55 (17) | 79 (5) |
| Unclassified | 12 (4) | 21 (8) | 33 (0) |

When the type of tumour is compared with the stage of spread (Table III) it becomes apparent that the majority of mucinous tumours were of an early stage, whereas there was a preponderance of inoperable tumours among the serous and unclassified types. The relative proportions of the different histological types of tumour in the three main stages are shown in Fig. 4, which also shows the proportion who died within 5 years of operation.

Although the better survival in the mucinous group is partly explained by the greater numbers in the earlier stages of development, it may be seen that there are differences in survival within each stage between the different types of tumour. Whilst the differences between serous and unclassified tumours are small, the differences between serous and mucinous are significant for unilateral intracapsular tumours ($\chi^2 = 6{\cdot}0$, $n = 1$, $P < 0{\cdot}02$) and for inoperable tumours ($\chi^2 = 5{\cdot}2$, $n = 1$, $P < 0{\cdot}05$), but do not reach significance in the intermediate group of operable tumours with spread beyond the ovarian capsule ($\chi^2 = 2{\cdot}17$, $n = 1$, $P < 0{\cdot}2$).

In some ways the survival figures are more disappointing than might have been hoped, for the apparent intactness of the ovarian capsule is clearly illusory. Microscopic spread must have already occurred in nearly half the serous and in the majority of the unclassified carcinomas which at operation were thought to be unilateral and intracapsular.

Serous and unclassified cancers behave very similarly at each stage, and the

FIG. 4.—The numbers of tumours of each histological type in each stage. The hatched areas give the number of patients who died within 5 years of operation.

results suggest very strongly that the inherent malignancy of these tumours is of overriding importance in their prognosis at every stage.

*The histological grade of the tumour*

The distribution of the cases according to their grade is shown in Table IV.

TABLE IV.—*The Distribution of Cases and Their 5-Year Survival According to Grade*

| Grade | Total at operation | 5-year survivors | percentage |
|---|---|---|---|
| 1 | 67 | 53 | 79 |
| 2 | 116 | 45 | 39 |
| 3 | 136 | 16 | 12 |

The differences in 5-year survival between the three groups is highly significant ($\chi^2 = 87{\cdot}2, n = 2, P < 0{\cdot}001$).

It would have been possible, by using different criteria, to have a more equal distribution of cases. But the relative paucity of cases in grade I and the excess of cases in grade 3 merely underlines the fact that carcinoma of the ovary is a highly malignant tumour, from which relatively few women survive.

Comparison of the grade of the tumour with the stage of the spread revealed, perhaps not surprisingly, that with few exceptions the more advanced the tumour, the less well differentiated it was. When the type of tumour was compared with grade, it was found that the majority of mucinous tumours were of grade I and the majority of unclassified tumours of grade 3. The serous tumours, apart from 13 per cent of grade I, were divided equally between grades 2 and 3. This, once again, shows the favourable nature of mucinous tumours and the high malignancy of serous and unclassified ones.

*The mitotic count and nuclear atypicality*

The system of grading described above depends on the pattern of the tumour seen, for the most part, under the low power of the microscope. Cellular atypicality and the frequency of mitosis, on the other hand, are features of the high power magnification. They were examined separately.

It was found on reviewing these carcinomas, that, apart from mitotic activity, the presence of nuclear atypicality is not a useful guide to the degree of malignancy. The groupings it encourages are few and there is a large indistinct borderland between the different groups. The well differentiated tumours are made up of cells with small nuclei, which are regular in shape and have inconspicuous nucleoli, and the few anaplastic tumours are composed of extremely bizarre cells with gross cellular atypia. All the tumours between these two extremes have minute gradations from the small and regular to the large reticular nucleus with a pronounced nucleolus. Accurate measurement of the size of the nucleolus, and its properties, shown by various histochemical techniques, have been discussed by Taylor and Long (1955), and have been found in their hands to be useful indicators of malignancy. But the techniques and methods required make it impossible for such methods ever to be used routinely, and without them the accurate assignment of tumours to different groups is impossible.

Counting the number of mitoses per high power field allows a much greater degree of precision than can be reached by considerations of atypicality. It was shown by Evans (1926) that tissues can remain unfixed for up to 24 hours without affecting the mitoses seen, so that even the interior of large malignant ovarian cysts gives a representative picture of the mitotic activity. By using such a system of counting, one can be confident of a greater degree of accuracy in assigning the tumours to four groups depending on their mitoses than into three groups based on the degree of nuclear atypicality.

Mitotic activity in ovarian carcinomas is not striking. In this study few tumours were found which had more than five mitoses per high power field, and very few had more than eight. The striking feature was the lack of mitoses even in poorly differentiated cancers. This observation was confirmed by Van Orden *et al.* (1966) who observed " a surprising finding was the paucity of tumours which had shown an increased number of mitoses . . . Only 13 per cent had mitoses as a prominent feature ". It conflicts, however, with the reports of Novak and Woodruff (1967), which appeared after our study had been largely completed. In the former study, the only one which has given numerical expression to an appraisal of mitotic activity in ovarian carcinomas, there was agreement with our observations that the number of mitoses correlated with the patient's chance of survival. In contrast with our findings and those of Van Orden *et al.* (1966), Novak and Woodruff reported a nigh number of mitoses in most tumours. It is probable that the procedure adopted was different from our method, where only cells in prophase and metaphase were counted, and, more important, where an average count of several fields in the most active area was taken. It is obviously easy to arrange a section in such a way that a particular field contains many more than the average number of mitoses for the tumour, and to count the number of of mitoses in the worst field. This would have been an alternative approach to the investigation, and probably the one adopted by Novak and Woodruff.

It was found that the presence of an average of three or more mitoses per high power field (see Fig. 5), gave a uniformly poor prognosis:

| Mitoses | Cases | 5-year survivors |
|---|---|---|
| 3/h.p.f. | 46 | 2 |
| 4/h.p.f. | 40 | 3 |
| 5 or more/h.p.f. | 36 | 3 |

Accordingly patients having tumours with three or more mitoses per high power field were amalgamated into one group, and when the tumours were classified in this way the following results were obtained:

| Mitoses | Cases | 5-year survivors | Percentage |
|---|---|---|---|
| $<1$/h.p.f. | 47 | 45 | 96 |
| 1/h.p.f. | 89 | 45 | 51 |
| 2/h.p.f. | 61 | 15 | 25 |
| $>2$/h.p.f. | 122 | 8 | 7 |

The 5-year survival figures for the patients with tumours of three or more mitoses was significantly worse than those in patients with two ($\chi^2 = 12{\cdot}03$, $n = 1$, $P < 0{\cdot}001$). Even with two mitoses the majority of patients died, but with only one mitosis the numbers were almost evenly divided, and with an average of less than one mitosis as in Fig. 6, almost all the patients lived. The difference in survival of patients with two mitoses and with one mitosis is highly significant ($\chi^2 = 10{\cdot}2$, $n = 1$, $P < 0{\cdot}01$); so also is the difference between one and less than one mitosis ($\chi^2 = 154, n = 1, P < 0{\cdot}001$).

When the mitotic count was compared with the other ways of assessing the tumours good correlations occurred.

(i) With regard to the stage reached at operation it was found that the unilateral intracapsular tumours had one, or less than one mitosis per high power field in 71 per cent of the cases. The operable tumours in stages IB to IIIA were fairly evenly spread, although with a relative lack of cases with less than one mitosis per high power field. In the highly malignant inoperable group, the majority showed three or more mitoses per high power field.

Evaluation of mitoses may also be useful in the cases of doubtful or borderline malignancy. Only two women died whose tumours had less than one mitosis per high power field, and in both cases the tumours were clinically malignant with spread.

(ii) A comparison of the mitotic count with the histological type of the tumour largely accounts for the superior survival figures of patients with mucinous carcinomas. Forty-five per cent had less than one mitosis per high power field, and only a quarter had two or more. By contrast, 43 per cent of serous, and 61 per cent of unclassified tumours had three or more mitoses per high power field. Classification by mitotic activity also explains the relatively benign behaviour of the adenoacanthoma (Fig. 2), since all in this series had few mitoses.

(iii) There is, not surprisingly, very great interdependence between the mitotic count and the grade of the tumour. They are, after all, two ways of expressing the histological malignancy of a carcinoma. In our series it was found that there were 245 patients in Grade 1 and Grade 3, and 30 of them behaved anomalously—that is, they died of tumours of Grade 1 or lived with tumours of Grade 3. There were on the other hand 224 patients whose tumours had an average of less than one or more than two mitoses per high power field, and only 10 of them behaved anomalously. The intermediate zone is larger when classifying by mitoses but from these results it seems that that disadvantage is more than outweighed by the much greater accuracy in prognostication.

### CONCLUSIONS

This survey has shown that the prognosis in carcinoma of the ovary is affected by the stage, type, grade and mitotic activity of the tumour.

(i) The stage of the tumour at operation: This has been shown to be highly significant in survival, and radiotherapy and chemotherapy hold out little hope if not all visible tumour is resected at the time of operation. Since resectability is a crucial factor in the patient's survival this has been given due weight by adopting a system of staging based on the operability of the tumour.

The other point which this staging system has emphasised is the importance of the intactness of the ovarian capsule. It is to some extent illusory, since there must have been microscopic penetration in the women with unilateral intracapsular tumours who died of carcinoma of the ovary. Nevertheless it is the only stage which carries a better than 50 per cent chance of survival, so it is important that women whose tumours are of this stage should be marked out as having a better than average prognosis.

(ii) The histological type of the tumour: The discussion in this survey has been centred on the relatively favourable mucinous carcinomas, although mention has also been made of those endometrioid carcinomas whose low grade is signalled by the development of squamous metaplasia. The demarcation of these tumours, however, only serves to emphasise the bleak outlook for the majority of patients with ovarian carcinoma, for most tumours are serous or unclassified and they are usually highly malignant.

(iii) The histological grade of the tumour: The histological grade has been shown to be related, to a great extent, to the stage of spread, and to a lesser extent to the histological type. It is revealed as a reliable indicator of the patient's prognosis—in fact a more sensitive means of discrimination than the stage of spread. Admittedly, the largest group of women had grade 3 tumours and only 21 per cent had grade 1 tumours, but most women with carcinoma of the ovary do die, and any attempt to balance the numbers by adopting different criteria for the various grades would only have diminished the usefulness of the distinctions between them.

(iv) The mitotic count: There is a highly significant degree of correlation between the mitotic count and survival. It corresponds closely with the grade of the tumour, and correlates with the stage, and to some extent with the type of the tumour. Although patients with mucinous carcinomas do better than those with serous or unclassified tumours for all mitotic counts, when adjustments are made for the generally earlier stage of mucinous carcinomas this superiority disappears. Thus, by using the mitotic count one may assess the malignant potential of a tumour without reference to its histological type. This has obvious advantages in a field as confused as the histological typing of ovarian carcinomas is at the moment. It also evades the difficulties inherent in having a class of unclassified tumours.

Comparison of the mitotic count with grading shows that to some extent they produce similar results since they are both ways of assessing the rate of growth of a tumour. However, a third way of assessing malignancy—the degree of cellular atypicality apart from mitoses—was not found helpful in this survey.

A mitotic count has the advantage over grading that it is easy to put into practice and is readily reproducible from time to time and person to person. Furthermore, it does not need previous agreement on the criteria for different grades (a problem which, as has been shown, bedevils grading). Both grading and mitotic activity give a fairly reliable guide to the behaviour of the majority of tumours (those in grades 1 and 3, and those with less than one or more than two mitoses) but the guidance from the mitotic count has the advantage that it is surer and less liable to prove fallible. Using the count of mitoses it is possible to predict with a good degree of accuracy the likely outcome for any woman with an ovarian carcinoma.

Our thanks are due to Dr. Magnus Haines of the Hospital for Women, Chelsea, and to Dr. P. C. Meyer of the Whittington Hospital, for allowing access to their materials and records. This work was in part supported by a Cancer Research Campaign grant.

### REFERENCES

Allan, M. S. and Hertig, A. T.—(1949) *Am. J. Obstet. Gynec.*, **58**, 640.
Barzilai, Gemma—(1943) ' Atlas of Ovarian Tumours '. New York (Grunne and Stratton, Inc.).
Bloom, H. J. G. and Richardson, W. W.—(1957) *Br. J. Cancer*, **11**, 359.
Cariker, Mildred and Dockerty, M. B.—(1954) *Cancer. N.Y.*, **7**, 302.
Carlin, G. J. and Frodey, R. J.—(1957) *Obstet. Gynec., N.Y.*, **9**, 71.
Counseller, V. S.—(1940) *Am. J. Surg.*, **49**, 284.
Cutler, S. J., Ederer, F., Griswold, M. H. and Greenberg, R. A.—(1960) *J. natn. Cancer, Inst.*, **24**, 541.
Czernobilsky, B., Silverman, B. B. and Enterline, H. T.—(1970) *Cancer, N.Y.*, **25** 762.
Davis, B. A., Latour, J. P. A. and Philpott, N. W.—(1956) *Surgery Gynec. Obstet.*, **102**, 565.
Evans, N.—(1926) *Archs Path.*, **1**, 894.
Ford, F. A.—(1928) *Am. J. Obstet. Gynec.*, **16**, 1.
Gardiner, G. A. and Slate, J.—(1956) *Am. J. Obstet. Gynec.*, **70**, 554.
Guerriero, W. F. and Spiedel, T.—(1963) *Am. J. Obstet. Gynec.*, **86**, 85.
Henderson, D. N. and Bean, J. L.—(1957) *Am. J. Obstet. Gynec.*, **73**, 657.
Heyman, J.—(1932) *Acta radiol.*, **13**, 329.
Keettel, W. C., Fog, M. R., Longnecker, D. S. and Latourette, H. B.—(1966) *Am. J. Obstet. Gynec.*, **94**, 766.
Kent, S. W. and McKay, D. G. —(1960) *Am. J. Obstet. Gynec.*, **80**, 430.
Kerr, H. D. and Elkins, H. B.—(1954) *Am. J. Roentg.*, **66**, 184.
Long, Margaret E. and Taylor, H. C.—(1964) *Am. J. Obstet. Gynec.*, **90**, 936.
Malloy, J. J., Dockerty, M. B., Welch, J. S. and Hunt, A. B.—(1965) *Am. J. Obstet. Gynec.*, **93**, 867 and 880.
Meigs, J. V.—(1940) *Surgery Gynec. Obstet.*, **71**, 44.
Montgomery, J. B. —(1948) *Am. J. Obstet. Gynec.*, **55**, 201.
Munnell, E. W., Jacog, H. W. and Taylor, H. C.—(1957) *Am. J. Obstet. Gynec.*,
Munnell, E. W. and Taylor, H. C.—(1949) *Am. J. Obstet. Gynec.*, **58**, 943.
Novak, E. R. and Nally, W. B.—(1957) *Obstet. Gynec., N.Y.*, **9**, 396.

NOVAK, E. R. AND WOODRUFF, J. D.—(1959) *Am. J. Obstet. Gynec.*, **77**, 632. (1967)—Novak's ' Gynecologic and Obstetric Pathology ', 6th edition. Philadelphia (W. B. Saunders Co.).
PARKER, T. M., DOCKERTY, M. B. AND RANDALL, L. M.—(1960) *Am. J. Obstet. Gynec.*, **80**, 417.
PEMBERTON, F. A.—(1940) *Am. J. Obstet. Gynec.*, **40**, 751.
PLATT, A. J., RUBENSTONE, A. I. AND HIRSCH, E. I.—(1962) *Am. J. Obstet. Gynec.*, **84**, 375.
POMERANCE, W., MOLTZ, A. AND HALL, J. E.—(1966) *Am. J. Obstet. Gynec.*, **96**, 418.
RATZKOVSKI, E. AND HOCHMAN, A.—(1963) *Cancer, N.Y.*, **16**, 1578.
RUBIN, P., GRISE, J. W. AND TERRY, R.—(1962) *Am. J. Roentg.*, **88**, 849.
SANTESSON, L. AND MARUBINI, G.—(1957) *Acta obstet. gynec. scand.*, **36**, 399.
SAGEN, E. A. AND HAKAMA, M.—(1964) *Natn. Cancer Inst. Monogr.*, **15**, 135.
SCHUELLER, E. F. AND KIROL, P. M.—(1966) *Obstet. Gynec., N.Y.*, **26**, 850.
SCULLY, R. E. AND BARLOW, J. F.—(1967) *Cancer, N.Y.*, **20**, 1405.
STONE, M. L., WEINGOLD, A. B., SALL, S. AND SONNENBLICK, B.—(1963) *Surgery Gynec. Obstet.*, **116**, 351.
STOWE, L. M.—(1955) *Cancer, N.Y.*, **8**, 446.
TAYLOR, H. C.—(1929) *Surgery Gynec. Obstet.*, **48**, 204.—(1959) *J. Obstet. Gynaec. Br. Commonw.*, **66**, 827.
TAYLOR, H. C. AND LONG, MARGARET E.—(1955) *Am. J. Obstet. Gynec.*, **70**, 753.
TURNER, J. C., REMINE, W. H. AND DOCKERTY, M. B.—(1959) *Surgery Gynec. Obstet.*, **109**, 198.
VAN ORDEN, DIANNA E., MCALLISTER, W. B., ZERNE, S. R. M. AND MORRIS, J. MCL.—(1966) *Am. J. Obstet. Gynec.*, **94**, 195.
WALTER, R. I., BACHMAN, A. L. AND HARRIS, W.—(1941) *Am. J. Roentg.*, **45**, 403.
WILLIS, R. A.—(1967) ' The Pathology of Tumours ', 4th edition. London (Butterworths) Chapter 29.


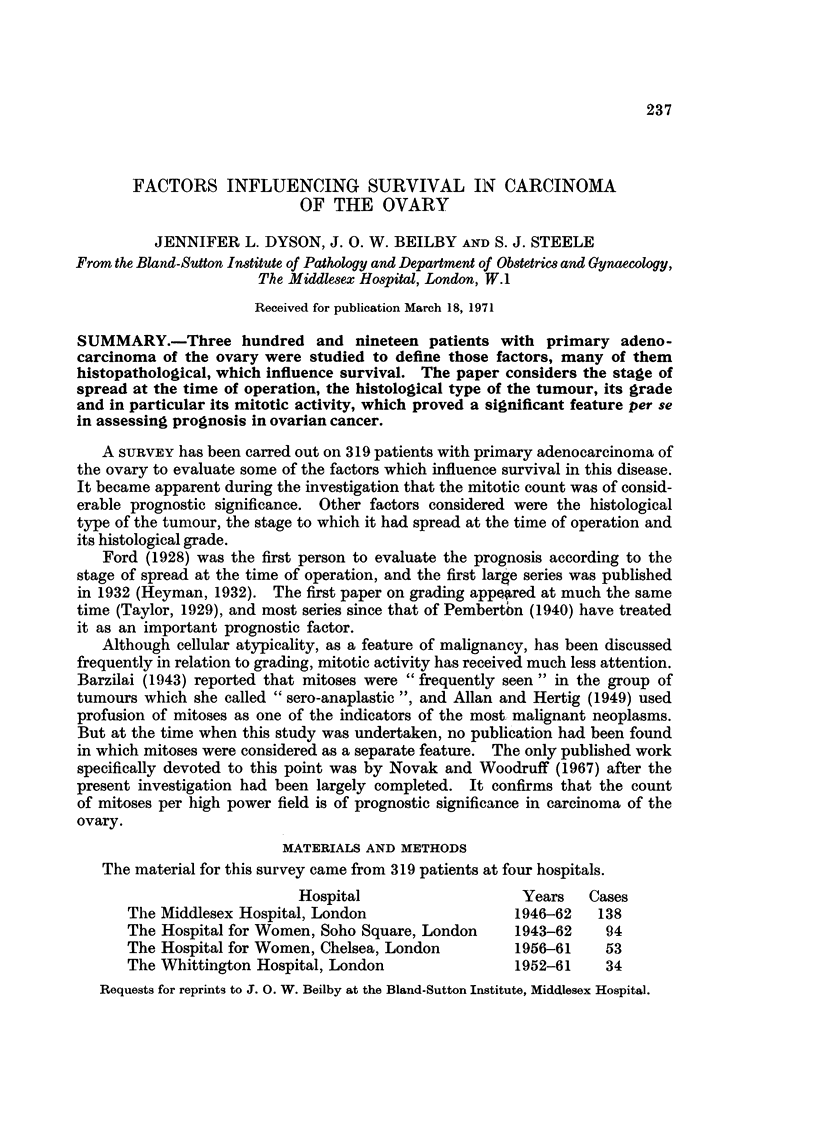

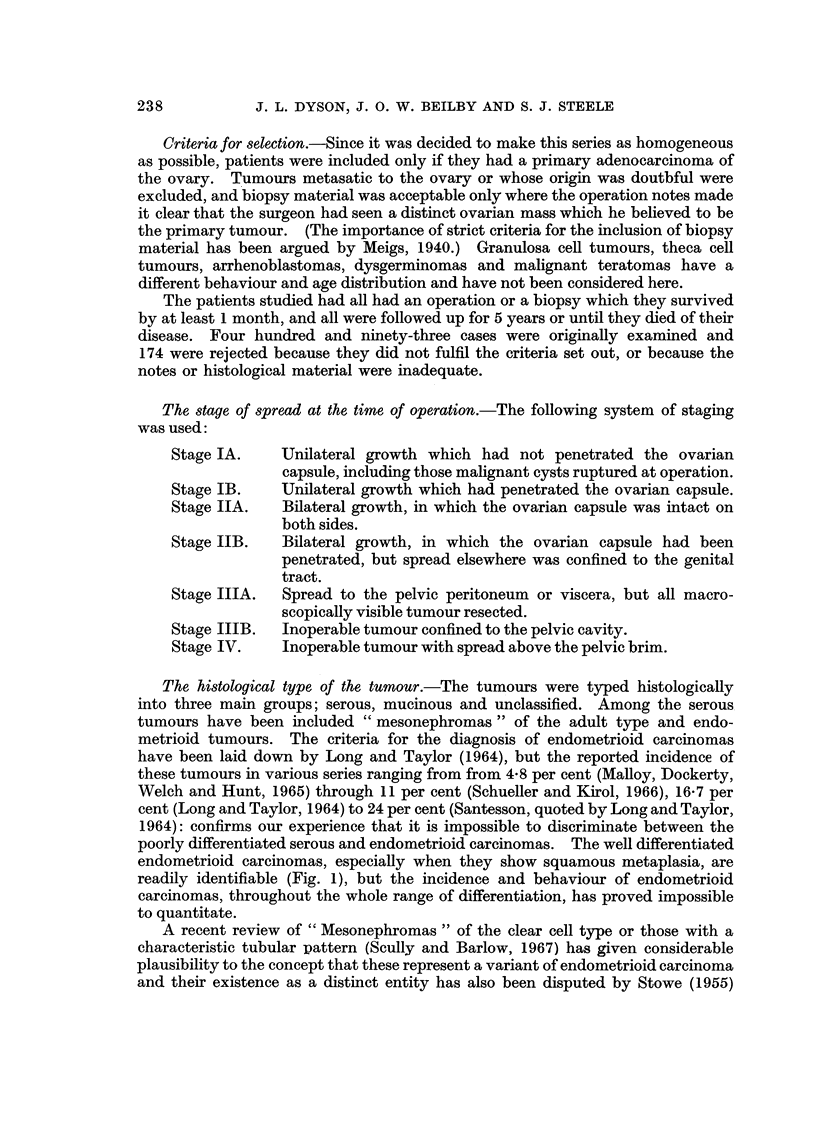

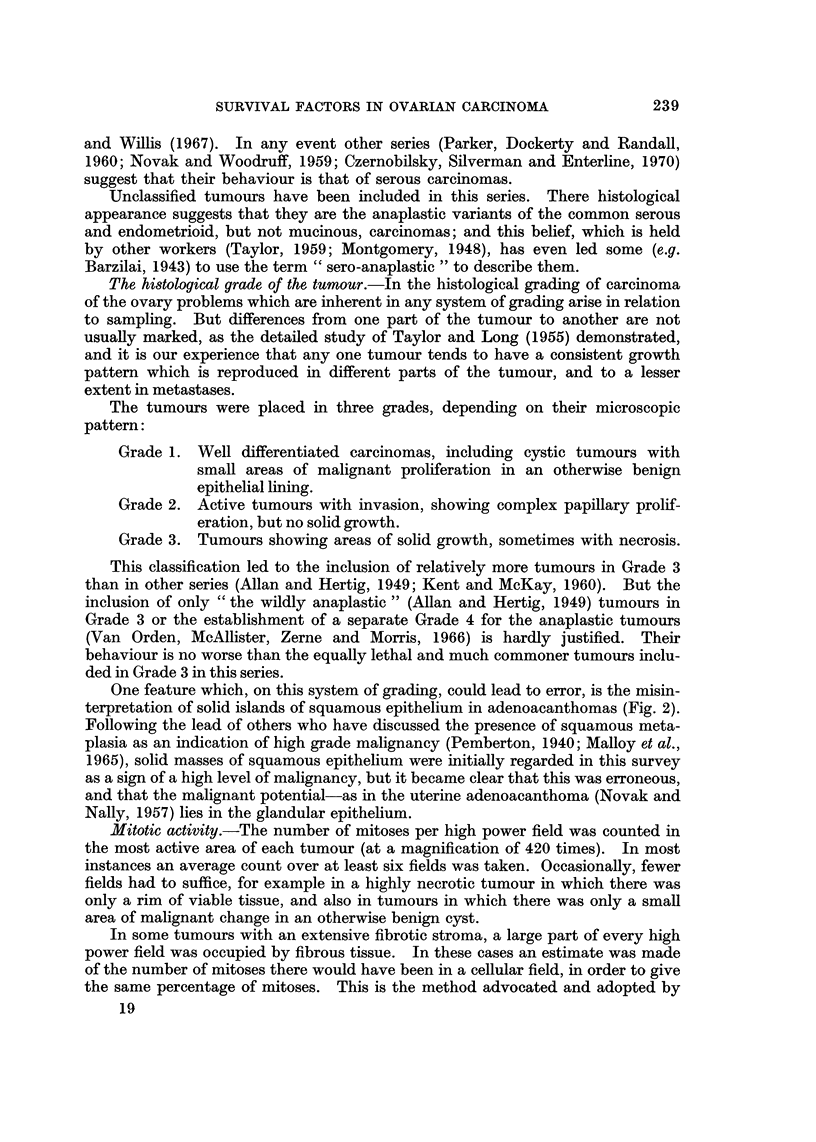

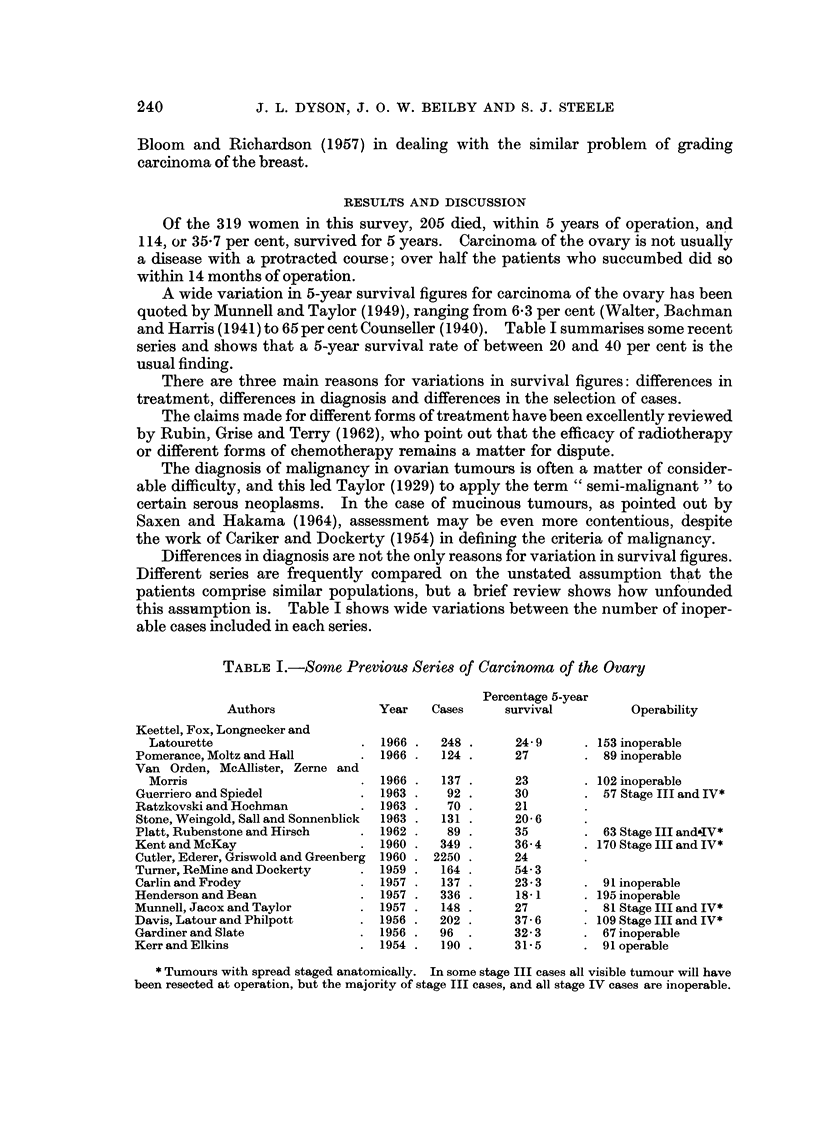

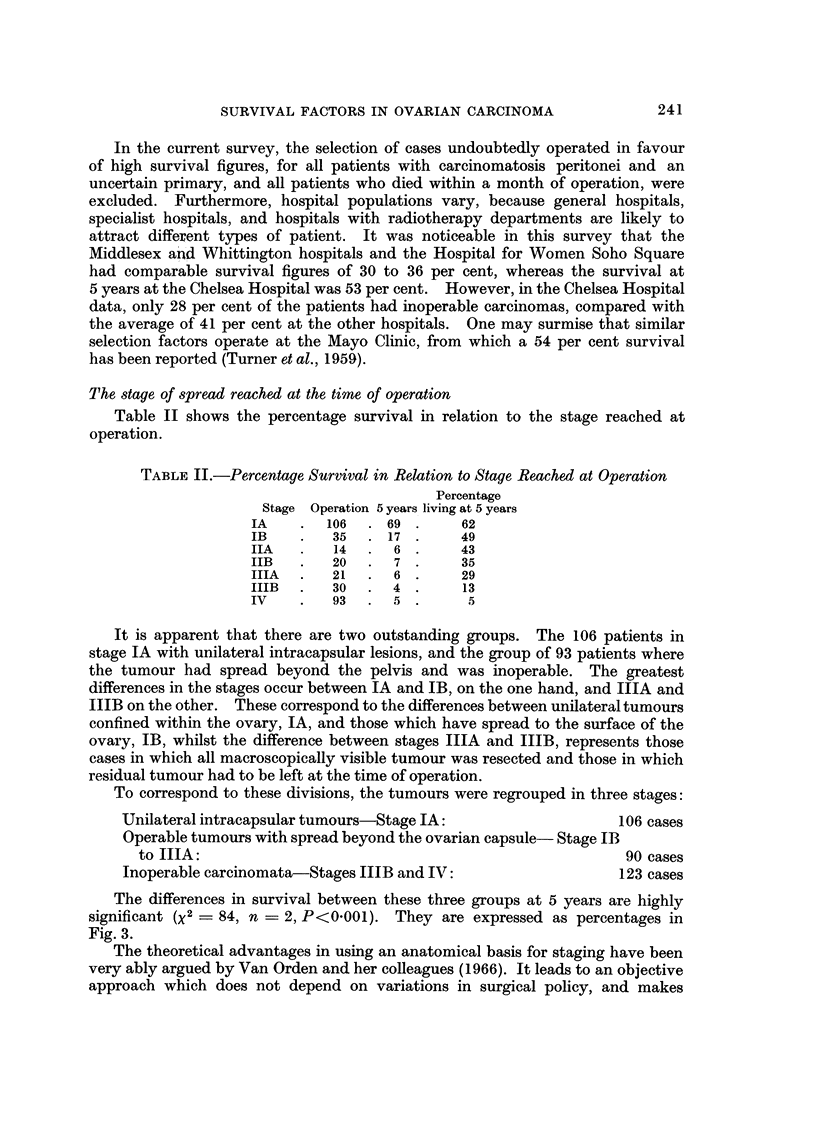

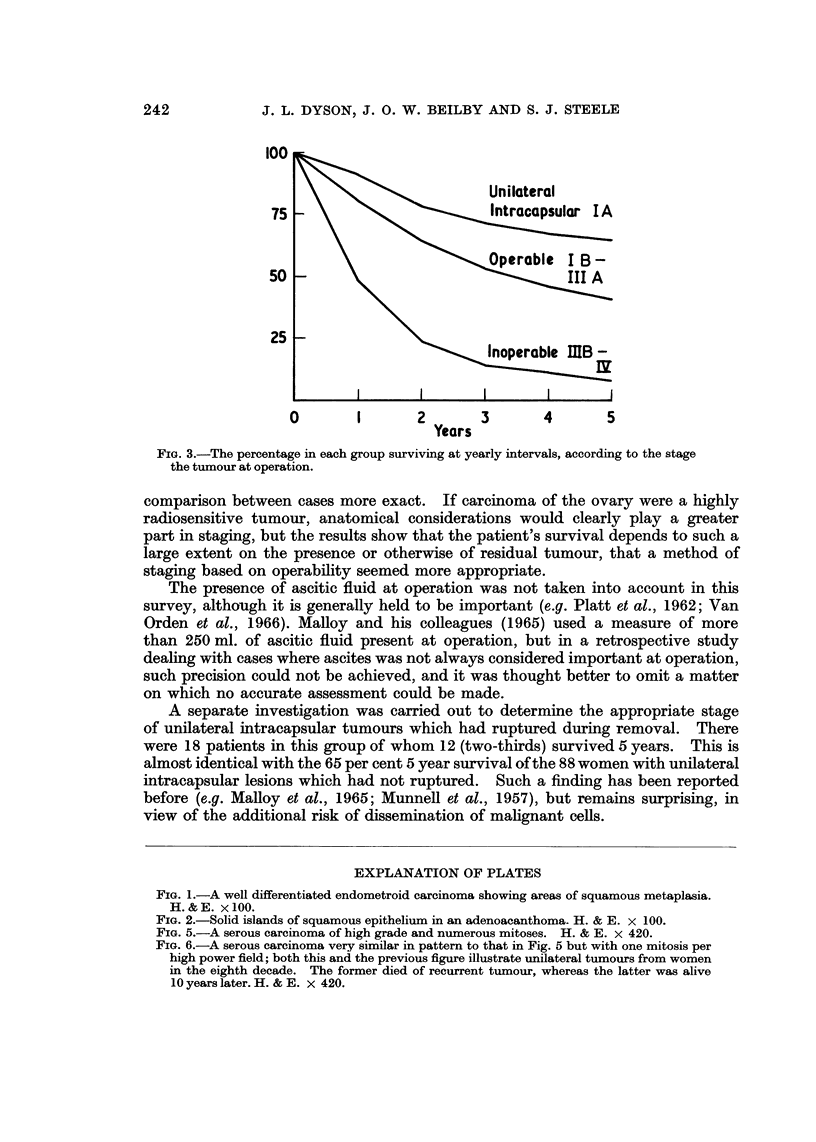

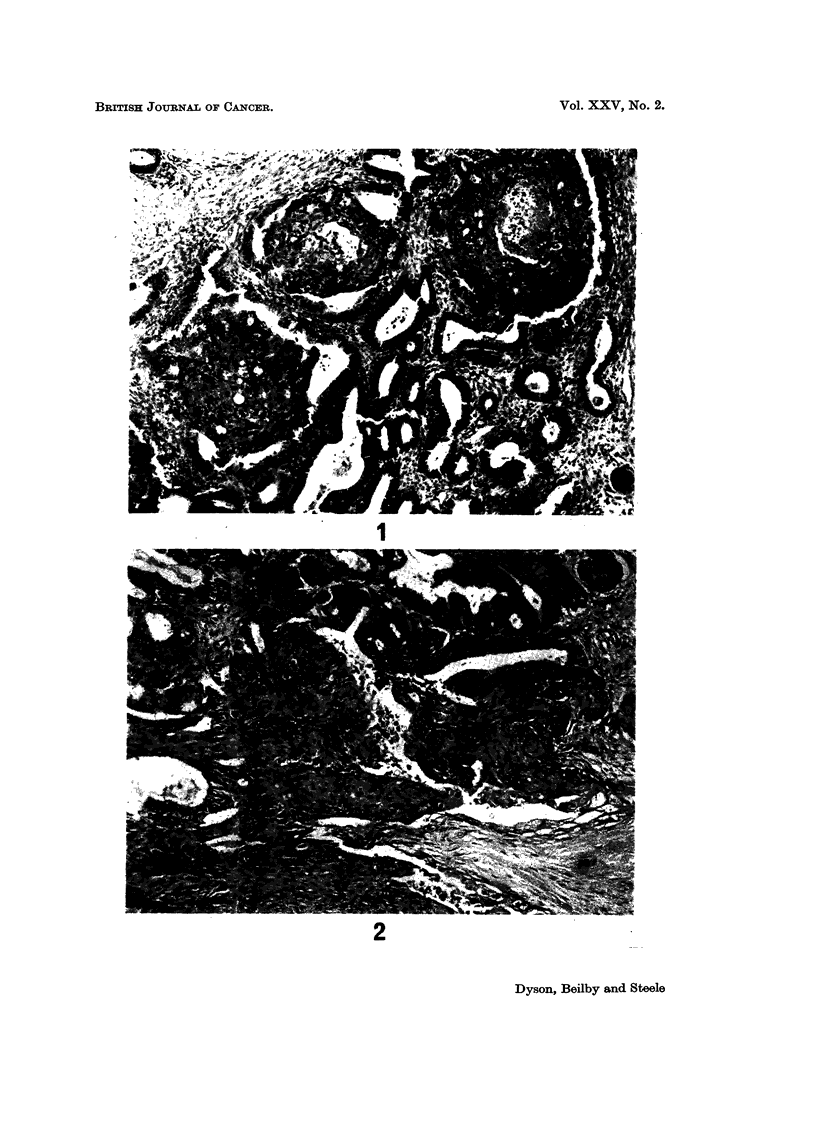

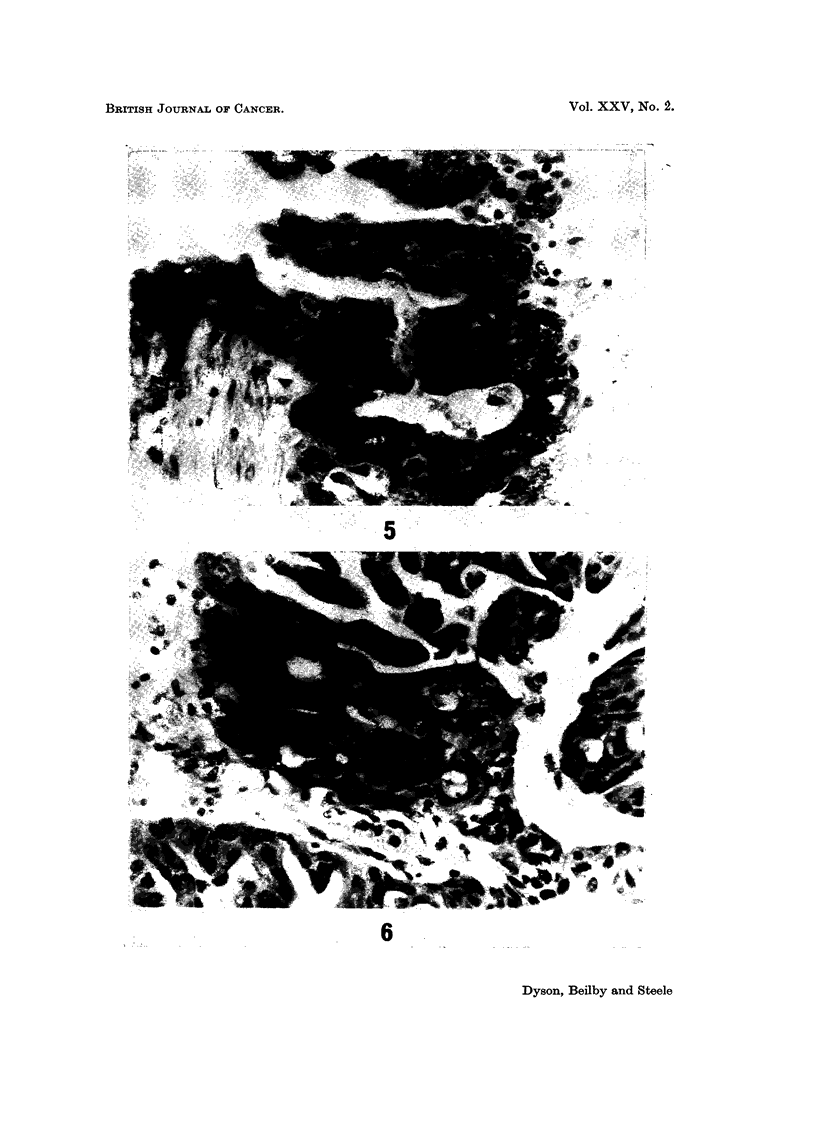

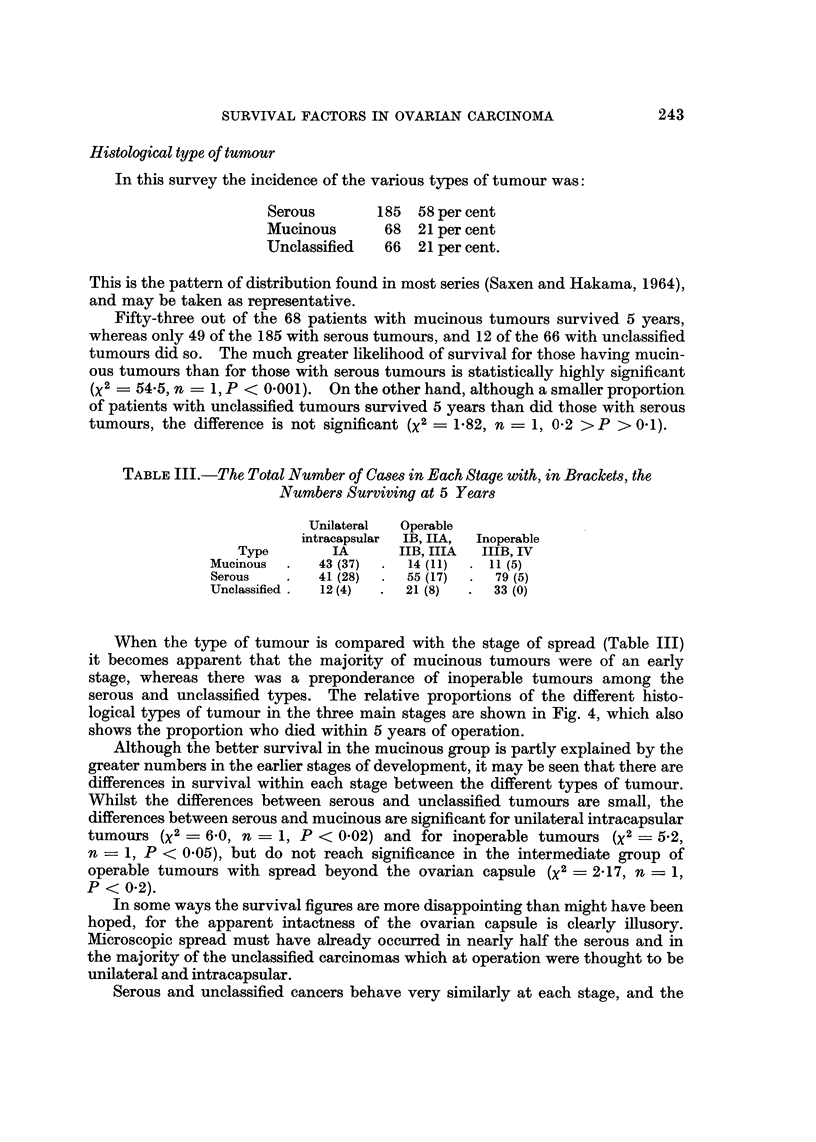

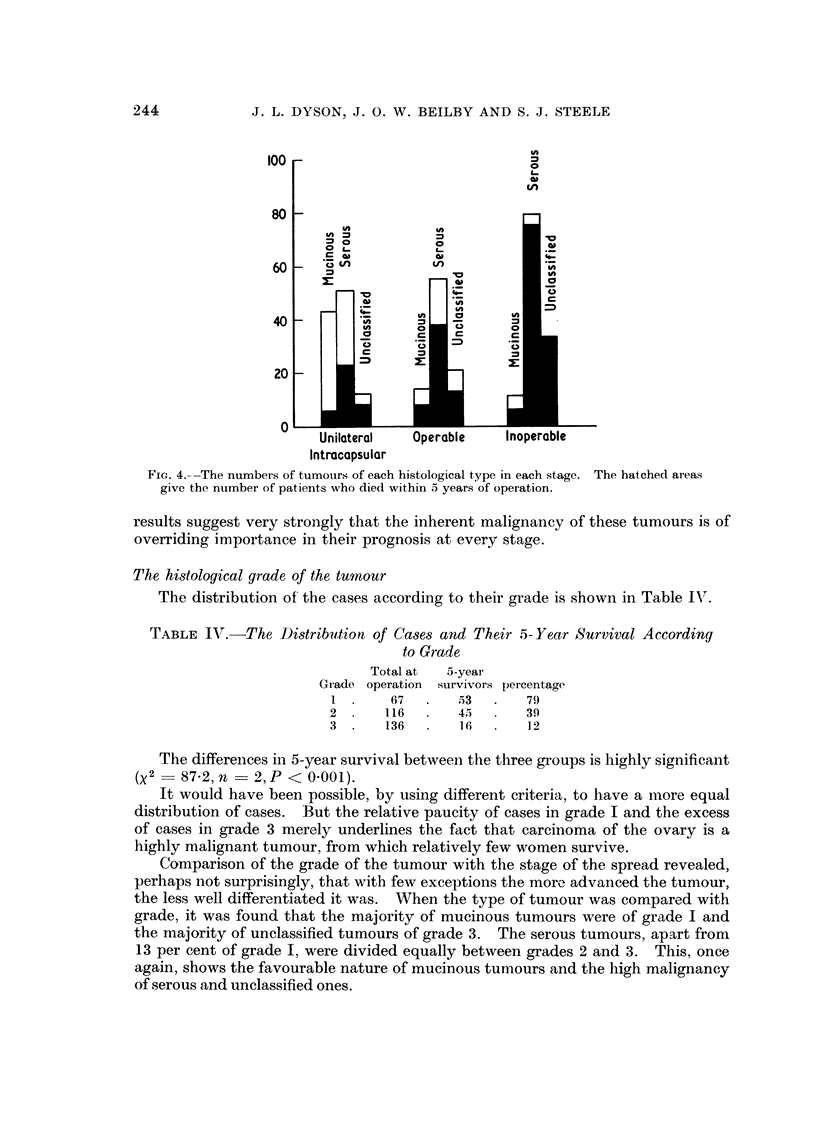

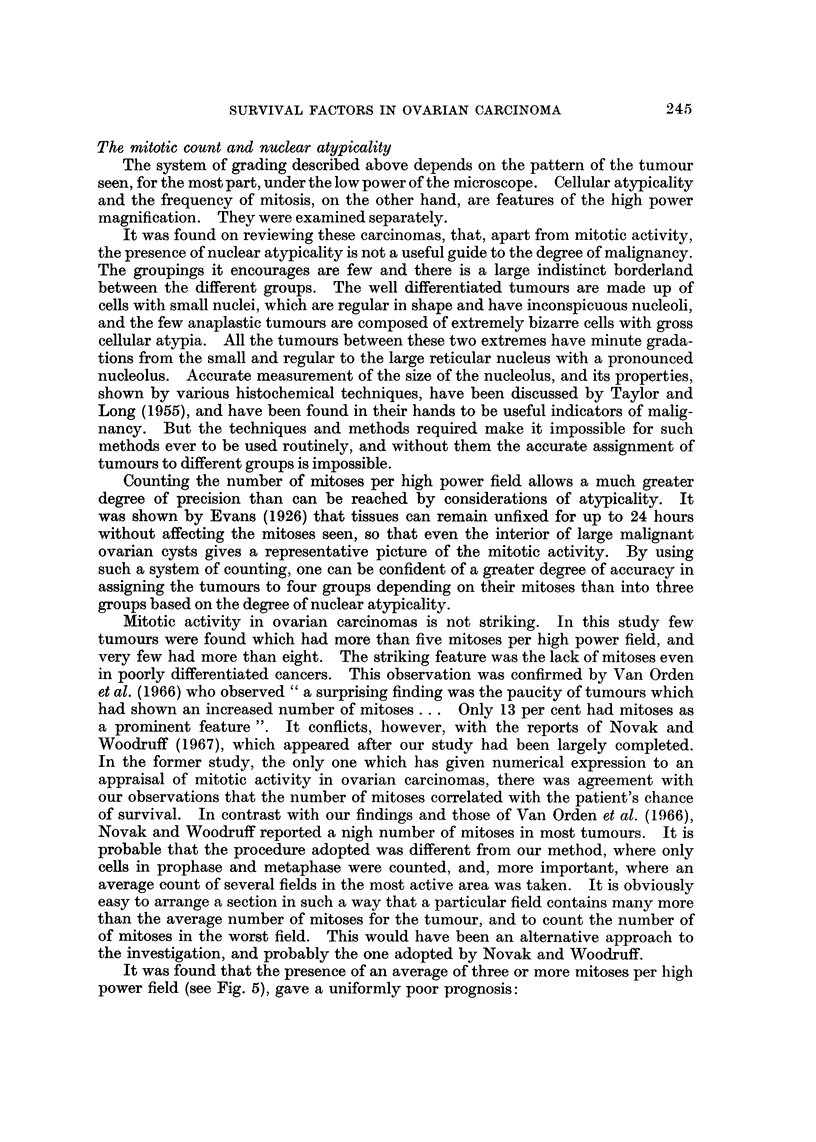

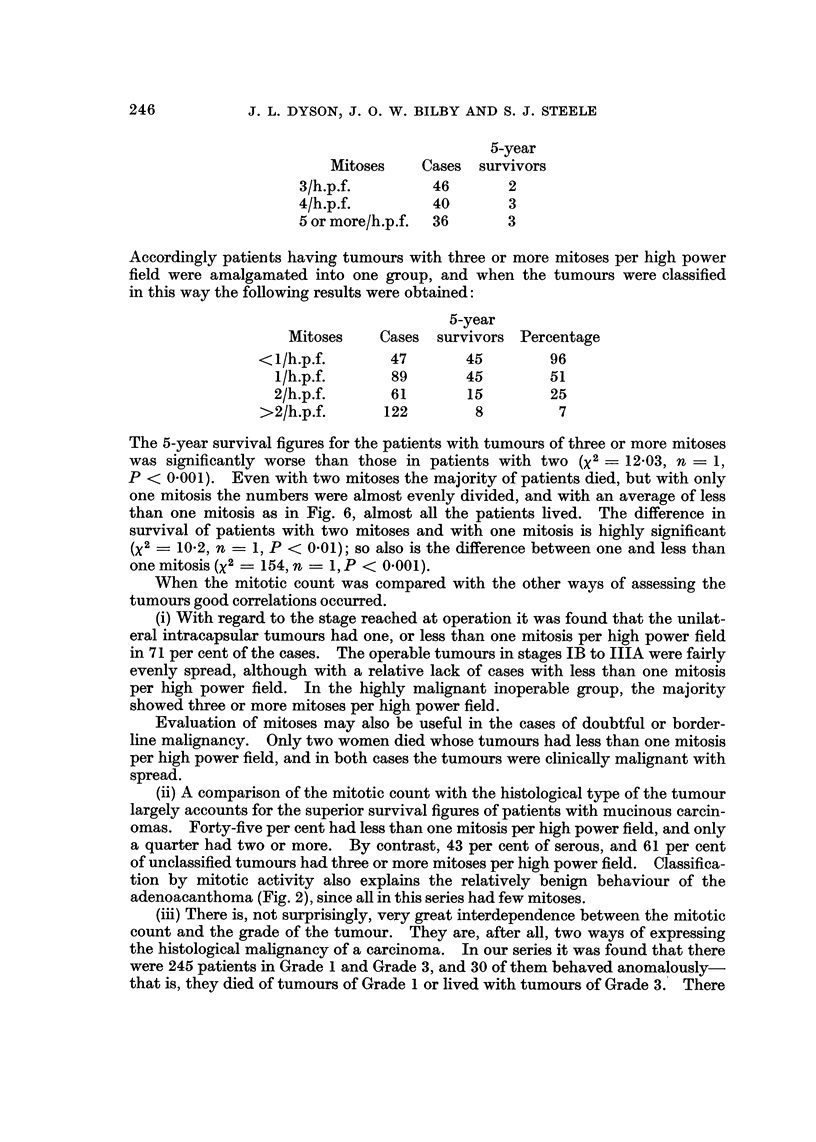

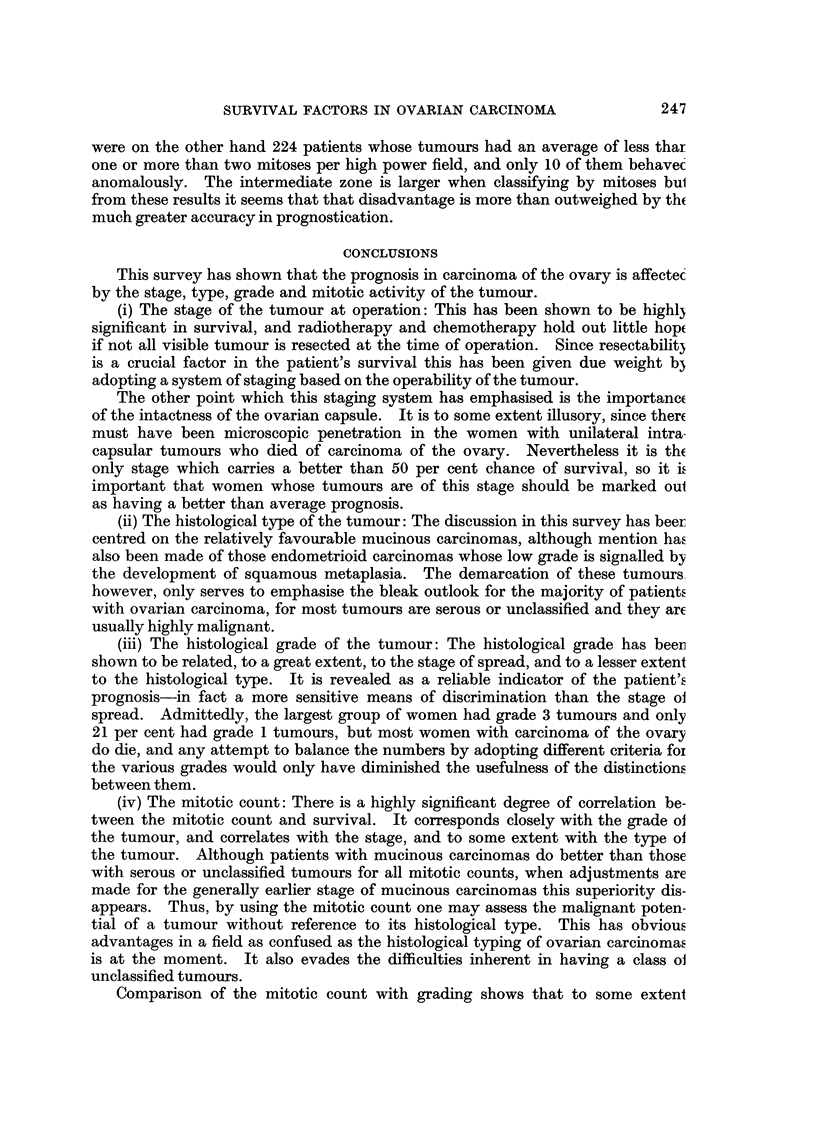

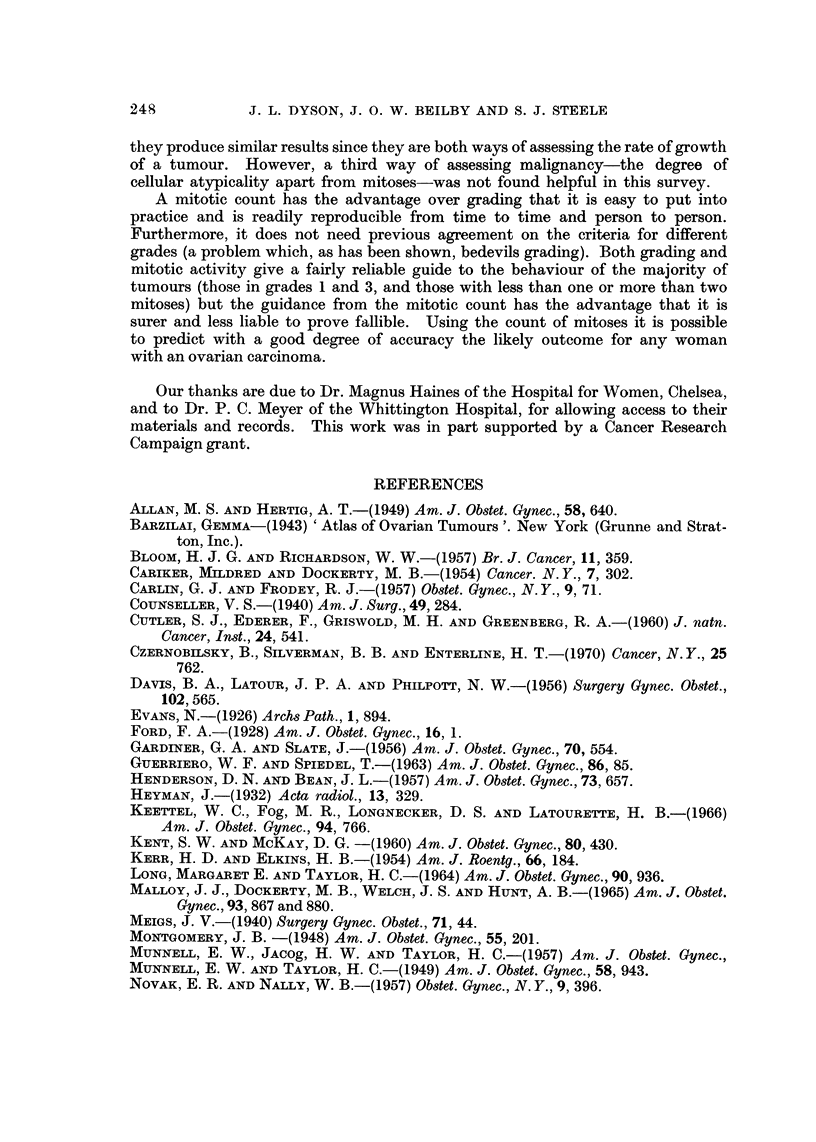

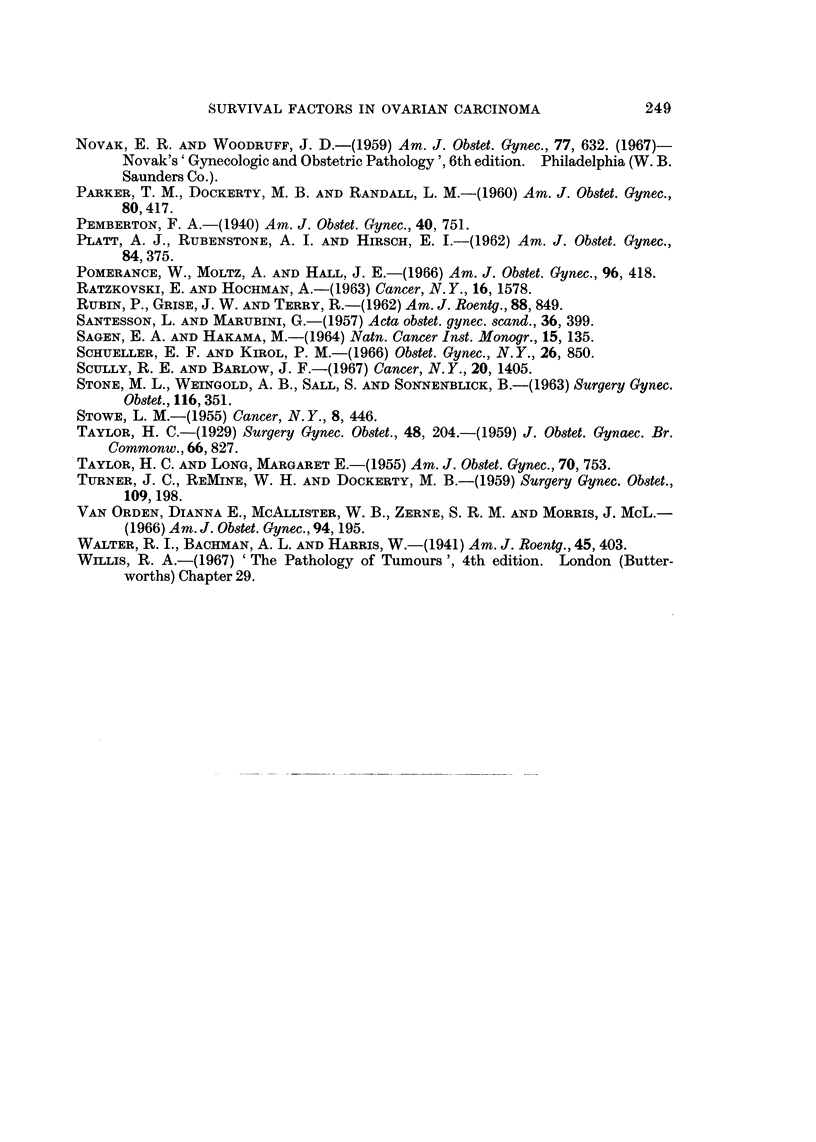


## References

[OCR_00751] CARIKER M., DOCKERTY M. (1954). Mucinous cystadenomas and mucinous cystadenocarcinomas of the ovary; a clinical and pathological study of 355 cases.. Cancer.

[OCR_00763] DAVIS B. A., LATOUR J. P., PHILPOTT N. W. (1956). Primary carcinoma of the ovary.. Surg Gynecol Obstet.

[OCR_00773] GARDINER G. A., SLATE J. (1955). Malignant tumors of the ovary.. Am J Obstet Gynecol.

[OCR_00774] HENDERSON D. N., BEAN J. L. (1957). Results of treatment of primary ovarian malignancy.. Am J Obstet Gynecol.

[OCR_00782] KERR H. D., ELKINS H. B. (1951). Carcinoma of the ovary.. Am J Roentgenol Radium Ther.

[OCR_00779] Keettel W. C., Fox M. R., Longnecker D. S., Latourette H. B. (1966). Prophylactic use of radioactive gold in the treatment of primary ovarian cancer.. Am J Obstet Gynecol.

[OCR_00784] LONG M. E., TAYLOR H. C. (1964). ENDOMETRIOID CARCINOMA OF THE OVARY.. Am J Obstet Gynecol.

[OCR_00795] MUNNELL E. W., TAYLOR H. C. (1949). Ovarian carcinoma; a review of 200 primary and 51 secondary cases.. Am J Obstet Gynecol.

[OCR_00786] Malloy J. J., Dockerty M. B., Welch J. S., Hunt A. B. (1965). Papillary ovarian tumors. II. Endometrioid cancers and mesonephroma ovarii.. Am J Obstet Gynecol.

[OCR_00802] NOVAK E. R., WOODRUFF J. D. (1959). [Mesonephroma of the ovary; thirty-five cases from the Ovarian Tumor Registry of the American Gynecological Society].. Am J Obstet Gynecol.

[OCR_00807] PARKER T. M., DOCKERTY M. B., RANDALL L. M. (1960). Mesonephric clear cell carcinoma of the ovary: a clinical and pathoolgic study.. Am J Obstet Gynecol.

[OCR_00811] PLATT A. J., RUBENSTONE A. I., HIRSH E. I. (1962). Factors affecting prognosis in carcinoma of the ovary.. Am J Obstet Gynecol.

[OCR_00815] Pomerance W., Moltz A., Hall J. E. (1966). Factors influencing survival in ovarian carcinoma.. Am J Obstet Gynecol.

[OCR_00821] SAXEN E. A., HAKAMA M. (1964). END RESULTS STUDIES ON CANCER OF THE OVARY.. Natl Cancer Inst Monogr.

[OCR_00825] STONE M. L., WEINGOLD A. B., SALL S., SONNENBLICK B. (1963). Factors affecting survival of patients with ovarian carcinoma.. Surg Gynecol Obstet.

[OCR_00829] STOWE L. M. (1955). On the genesis of the so-called mesonephroma ovarii.. Cancer.

[OCR_00823] Scully R. E., Barlow J. F. (1967). "Mesonephroma" of ovary. Tumor of Müllerian nature related to the endometrioid carcinoma.. Cancer.

[OCR_00835] TAYLOR H. C., LONG M. E. (1955). Problems of cellular and tissue differentiation in papillary adenocarcinoma of the ovary.. Am J Obstet Gynecol.

[OCR_00831] TAYLOR H. C. (1959). Studies on the clinical and biological evolution of adenocarcinoma of the ovary.. J Obstet Gynaecol Br Emp.

[OCR_00837] TURNER J. C., REMINE W. H., DOCKERTY M. B. (1959). A clinicopathologic study of 172 patients with primary carcinoma of the ovary.. Surg Gynecol Obstet.

[OCR_00841] Van Orden D. E., McAllister W. B., Zerne S. R., Morris J. M. (1966). Ovarian carcinoma. The problems of staging and grading.. Am J Obstet Gynecol.

